# Reversible Severe Cardiomyopathy Associated With Supratherapeutic Vancomycin Levels: A Case Report

**DOI:** 10.7759/cureus.98948

**Published:** 2025-12-11

**Authors:** Moayad Alfayoumi, Mohamad Y Khatib, Ghada M Aboujassoum, Abdulqadir J Nashwan

**Affiliations:** 1 Pharmacy Department, Hamad Medical Corporation, Doha, QAT; 2 Critical Care Medicine Department, Hamad Medical Corporation, Doha, QAT; 3 Nursing and Midwifery Research Department, Hamad Medical Corporation, Doha, QAT

**Keywords:** hemodialysis, left ventricular thrombus, reversible cardiomyopathy, supratherapeutic drug levels, vancomycin, vancomycin toxicity

## Abstract

Vancomycin is a widely used glycopeptide antibiotic, and while nephrotoxicity and infusion-related reactions are well-established adverse effects, direct reversible cardiomyopathy associated with extreme vancomycin levels has not been previously reported in humans. We describe a 59-year-old woman treated for methicillin-resistant *Staphylococcus aureus* (MRSA) bacteremia who developed acute heart failure, hypotension, and severe left ventricular systolic dysfunction (ejection fraction 27%) while her vancomycin level exceeded 80 mg/L. Multiple left ventricular thrombi were also identified, likely representing a secondary complication of profound systolic dysfunction, with a possible contributory prothrombotic effect of supratherapeutic vancomycin levels. Extensive evaluation excluded ischemic, infectious, autoimmune, and other drug-induced causes of cardiomyopathy. As serial hemodialysis sessions gradually removed vancomycin, cardiac function improved, with the ejection fraction increasing to 44%. The temporal relationship, laboratory findings, and Naranjo scoring support a probable causal association between supratherapeutic vancomycin exposure and reversible cardiomyopathy. This case highlights a potentially underrecognized adverse effect. It emphasizes the importance of therapeutic drug monitoring and cardiac assessment when vancomycin levels become markedly elevated, especially in patients with impaired renal clearance.

## Introduction

Vancomycin is a glycopeptide antibiotic widely used as first-line therapy for invasive methicillin-resistant *Staphylococcus aureus* (MRSA) infections [1,2]. Its adverse effect profile is well established, with nephrotoxicity being one of the most clinically significant complications, attributed to oxidative stress, mitochondrial injury, and tubular necrosis described in multiple reports [3-9]. Infusion-related reactions, including vancomycin infusion reaction (formerly known as red man syndrome), are also recognized, typically resulting from non-IgE-mediated histamine release and occasionally presenting with cardiovascular instability during rapid administration [10-12]. Importantly, several known risk factors for vancomycin toxicity include low body weight, impaired renal function, and inconsistent or interrupted treatment exposure - conditions that heighten the risk of supratherapeutic accumulation when standard fixed doses (e.g., 1 g or 750 mg) are used in patients significantly below average adult weight. This is particularly relevant to the present case, in which the patient weighed only 40 kg, making the absolute doses received disproportionately high relative to her body mass.

Although cardiovascular events secondary to infusion reactions, such as vasospasm or Kounis syndrome, have been reported in the literature [13-19], there are no published human cases describing direct myocardial toxicity or reversible cardiomyopathy resulting from sustained supratherapeutic vancomycin levels. Experimental evidence from isolated working rat heart models has demonstrated that vancomycin can exert dose-dependent cardiotoxic effects at very high concentrations, including bradycardia and reduced coronary perfusion, independent of cholinergic or calcium-channel pathways [13]. These findings support the plausibility of cardiotoxicity but, until now, have not been observed clinically.

This report presents the first documented human case of reversible left ventricular (LV) systolic dysfunction associated with markedly elevated vancomycin concentrations. The case underscores a previously unrecognized potential adverse effect of vancomycin and highlights the importance of therapeutic drug monitoring (TDM) and cardiac assessment in patients with impaired renal clearance or prolonged exposure to supratherapeutic drug levels.

## Case presentation

A 59-year-old woman weighing 40 kg presented in May 2025 with MRSA bacteremia and was treated with multiple courses of intravenous vancomycin. Her therapy was frequently interrupted due to refusal of treatment and episodes of discharge against medical advice. Vancomycin was initiated with a 1,500-mg loading dose, followed by 1 g three times daily for several days. After an early treatment interruption, therapy was restarted with a 1 g stat dose followed by 750 mg three times daily for several more days. A second interruption subsequently occurred. Vancomycin was then reinitiated with a 1 g stat dose followed by 750 mg three times daily and continued until the time of clinical deterioration. No TDM was performed during these periods, despite the patient's low body weight and inconsistent treatment exposure - both recognized risk factors for toxicity.

In early June 2025, the patient was admitted to the medical intensive care unit (MICU) with sudden-onset dyspnea, profound fatigue, and hypotension (mean arterial pressure 50-55 mmHg). She denied chest pain. Physical examination revealed elevated jugular venous pressure (8 cm), bilateral lower-limb pitting edema, and bibasilar crackles. There was no rash, flushing, pruritus, or urticaria to suggest a vancomycin infusion reaction. Cardiac auscultation revealed a new S3 gallop. Electrocardiography demonstrated sinus tachycardia with nonspecific ST-T abnormalities.

A serum vancomycin level measured immediately on admission exceeded 80 mg/L, significantly surpassing the threshold associated with toxicity [3]. Vancomycin was discontinued. Transthoracic echocardiography (TTE) demonstrated new severe LV systolic dysfunction with an ejection fraction (EF) of 27% and multiple LV thrombi. Comparison with a prior baseline TTE performed in 2025, which showed a normal EF of 52%, confirmed the acute decline in cardiac function.

Over the subsequent several days, vancomycin levels persistently remained above 80 mg/L. During the same period, the patient developed acute kidney injury, with serum creatinine rising markedly from baseline, consistent with vancomycin-associated nephrotoxicity described in prior reports. Hemodialysis was initiated to enhance drug clearance. Following the first session, vancomycin levels declined to approximately 70 mg/L. Serial hemodialysis sessions over the following days resulted in a progressive reduction to approximately 23 mg/L. A repeat TTE performed after this decline demonstrated partial improvement in EF to 34%.

Throughout hospitalization, extensive investigations were performed to rule out alternative causes of cardiomyopathy. Cardiology evaluation and imaging revealed no evidence of ischemic heart disease. Infectious and inflammatory etiologies were excluded through negative viral serologies, regular inflammatory markers, and sterile blood cultures. There was no exposure to other cardiotoxic medications, no clinical features of severe sepsis, no emotional trigger to suggest Takotsubo cardiomyopathy, and no evidence of anaphylaxis or vancomycin infusion reaction.

As vancomycin levels normalized, the patient’s clinical status continued to improve. A follow-up TTE performed approximately one month later demonstrated EF recovery to 44%, supporting the reversibility of the myocardial dysfunction. LV thrombi persisted but decreased in size, and the patient remained on therapeutic anticoagulation. She was stepped down from the MICU to the medical ward in a stable condition.

A detailed overview of the treatment timeline, fluctuations in vancomycin levels, initiation of hemodialysis, and progressive improvement in EF is provided in Table 1, illustrating the temporal relationship supporting vancomycin-associated cardiotoxicity.

**Table 1:** Timeline of Clinical Events, Vancomycin Levels, and Echocardiographic Findings MICU: Medical Intensive Care Unit; HD: hemodialysis; LV: left ventricular; TDM: therapeutic drug monitoring; AKI: acute kidney injury; Cr: creatinine

Day	Event/Intervention	Vancomycin Level	Ejection Fraction (EF)	Notes
Day 1	Baseline transthoracic echocardiogram	-	52%	Normal LV function
Day 25	Vancomycin started (1500 mg LD → 1 g TID)	-	-	-
Days 26-29	Treatment interrupted (patient refusal)	-	-	No TDM performed
Day 30	Vancomycin restarted (1 g stat → 750 mg TID)	-	-	-
Days 36-41	Second treatment interruption	-	-	-
Day 42	Vancomycin restarted (1 g stat → 750 mg TID)	-	-	Continued until Day 51
Day 51	Acute decompensation → MICU admission	>80 mg/L	27%	Severe LV dysfunction + multiple LV thrombi
Days 51-55	Persistent supratherapeutic levels	>80 mg/L	-	AKI worsens (Cr ↑ to 235 mmol/L)
Day 56	Hemodialysis initiated	-	-	First HD session
Day 57	After the first HD session	70.1 mg/L	-	Partial drug clearance
Days 57-66	Serial HD sessions	Declining	-	-
Day 66	Repeat echocardiogram	22.7 mg/L	34%	Improving LV function
Day 72	Stepped down from the MICU to the ward	-	-	Clinically stable
Day 86	Follow-up echocardiogram	Undetectable/cleared	44%	Significant recovery

## Discussion

The clinical course of this patient provides compelling evidence for a probable association between supratherapeutic vancomycin levels and reversible cardiomyopathy. Although vancomycin is widely used for serious Gram-positive infections, its adverse effects are typically limited to nephrotoxicity, infusion-related reactions, and, less commonly, hypersensitivity-mediated cardiovascular events [1,3,10-12]. To date, there have been no previous human reports of direct myocardial toxicity associated with sustained supratherapeutic vancomycin exposure. The sharp decline in cardiac function in this case, coupled with recovery following drug clearance, supports a causal relationship.

Using the Naranjo Adverse Drug Reaction Probability Scale, the reaction was scored 7, consistent with a probable drug-induced adverse event. The temporal association was clearly demonstrated: the patient’s EF decreased significantly while vancomycin levels exceeded 80 mg/L and improved steadily as the concentration was lowered through hemodialysis. No alternative explanations for the cardiomyopathy were identified. Ischemic etiology was excluded by cardiology evaluation; infectious and inflammatory causes were ruled out with a negative workup; no other cardiotoxic medications were administered; and the patient exhibited no features suggestive of sepsis- or stress-induced cardiomyopathy. Similarly, an infusion-related reaction was unlikely because the patient lacked cutaneous manifestations, and the cardiomyopathy developed during sustained high drug concentration during active infusion [10-12,17-19].

Mechanistically, several pathways support the plausibility of vancomycin-induced myocardial dysfunction. Experimental studies using isolated rat hearts demonstrated that vancomycin at extremely high concentrations produces direct cardiotoxic effects, including bradycardia, reduced coronary perfusion, and impaired contractility, independent of parasympathetic activity or calcium-channel blockade [13]. These findings suggest that vancomycin may exert direct myocardial toxicity at elevated serum levels. Additional animal data show that vancomycin exposure increases oxidative stress and mitochondrial injury, mechanisms known to impair cardiac function [14]. Mitochondrial dysfunction, in particular, parallels the mechanism of anthracycline cardiotoxicity, where inhibition of electron transport leads to reduced adenosine triphosphate (ATP) synthesis, impaired contractility, and dose-dependent reversibility [15]. These pathways may collectively account for the acute, but reversible, decline in LV function observed in this patient.

Previous reports of vancomycin-associated cardiac complications have primarily involved infusion-related events such as Kounis syndrome, coronary vasospasm, or stress cardiomyopathy triggered by hypersensitivity reactions [17-19]. These cases differ significantly from the present report, which describes a delayed-onset cardiomyopathy during periods of elevated vancomycin concentration rather than an immediate hypersensitivity response. Unlike the previously documented events, our patient had no cutaneous symptoms, no chest pain, no eosinophilia, and no electrocardiographic features suggestive of vasospasm or myocardial ischemia.

Another notable feature was the development of multiple LV thrombi. Ventricular thrombus formation most commonly results from profound systolic dysfunction, and in this case, the marked reduction in EF likely created significant blood stasis - the primary driver consistent with Virchow’s triad [20]. While we discuss the possibility that vancomycin-associated oxidative stress could contribute to endothelial injury and a prothrombotic milieu, this should be regarded as a secondary hypothesis and an area warranting further investigation, rather than a definitive mechanism. The coexistence of blood stasis, systemic illness, and potential endothelial effects provides a plausible framework but requires additional experimental evidence to establish potential causality [20].

This case represents the first documented human report of reversible cardiomyopathy associated with markedly elevated vancomycin levels. The patient’s dramatic improvement in EF after drug clearance underscores the importance of timely TDM, particularly in individuals with fluctuating renal function or interrupted dosing patterns. It also suggests that echocardiographic surveillance may be warranted when vancomycin concentrations exceed 50-60 mg/L, especially if accompanied by clinical signs of cardiovascular compromise.

## Conclusions

This case documents a probable association between sustained supratherapeutic vancomycin levels and reversible severe LV systolic dysfunction. Cardiac function improved following drug discontinuation and hemodialysis-facilitated clearance, supporting a direct toxic effect. Rigorous TDM is essential, particularly in patients with low body weight or renal impairment. Cardiac assessment should be considered when vancomycin concentrations exceed approximately 50-60 mg/L to allow early detection and prevention of serious cardiotoxicity.
